# Peripheral Blood Mononuclear Cells Oxidative Stress and Plasma Inflammatory Biomarkers in Adults with Normal Weight, Overweight and Obesity

**DOI:** 10.3390/antiox10050813

**Published:** 2021-05-20

**Authors:** Margalida Monserrat-Mesquida, Magdalena Quetglas-Llabrés, Cristina Bouzas, Xavier Capó, David Mateos, Lucía Ugarriza, Josep A. Tur, Antoni Sureda

**Affiliations:** Research Group in Community Nutrition and Oxidative Stress, University of the Balearic Islands-IUNICS, Health Research Institute of Balearic Islands (IdISBa), and CIBEROBN (Physiopathology of Obesity and Nutrition), E-07122 Palma, Balearic Islands, Spain; margalida.monserrat@uib.es (M.M.-M.); m.quetglas@uib.es (M.Q.-L.); cristina.bouzas@uib.es (C.B.); xavier.capo@uib.es (X.C.); david.mateos@uib.es (D.M.); lugarriza@ibsalut.caib.es (L.U.); antoni.sureda@uib.es (A.S.)

**Keywords:** obesity, antioxidants, oxidative stress, inflammation, immune cells, PBMC

## Abstract

**Background:** Obesity is an important pathology in public health worldwide. Obese patients are characterized by higher cardiovascular risk and a pro-inflammatory profile. **Objective:** To assess the oxidative stress in peripheral blood mononuclear cells (PBMCs) and inflammatory biomarkers in plasma in adults with normal weight, overweight and obesity. **Methods:** One hundred and fifty adults (55-80-years-old; 60% women) from the Balearic Islands, Spain, were recruited and classified according to body mass index (BMI). Anthropometric measurements were carried out, fasting blood samples were collected and plasma and PBMCs were obtained. Biochemical parameters, hemogram, antioxidant enzyme activities and protein levels, reactive oxygen species production (ROS), malondialdehyde (MDA), and cytokine (tumour necrosis factor, TNFα, and interleukin 6, IL-6) levels were measured. **Results:** Glycaemia, triglyceridemia, abdominal obesity, and waist-to-height ratio (WHtR) were higher, and HDL-cholesterol was lower in obese patients. MDA and TNFα plasma levels were higher in the obese compared to normal-weight group, while the levels of IL-6 were higher in both obese and overweight subjects with respect to normal-weight peers. The activities of all antioxidant enzymes in PBMCs as well as the production ROS progressively increased with BMI. The protein levels of catalase in PBMCs were higher in obese and glutathione reductase in obese and overweight subjects compared to normal-weight peers. No other differences were observed. **Conclusion:** The current results show that overweight and obesity are related to an increase in pro-oxidant and proinflammatory status in plasma and PBMCs. The studied biomarkers may be useful for monitoring the progression/reversal of obesity.

## 1. Introduction

Obesity is considered a clear threat to public health worldwide, and, together with overweight, constitutes one of the main factors that promote non-communicable diseases (NCDs) [1]. Direct associations have been evidenced between obesity and high blood pressure, type 2 diabetes mellitus, cardiovascular diseases, musculoskeletal disorders and several cancers.

Obesity is characterized by an increase in oxidative stress and a pro-inflammatory state [2]. The pathogenesis of this disorder is characterized by the existence of an imbalance in the oxidative/antioxidant state and a low-grade subclinical inflammatory state that results in an increased risk of atherosclerotic and diabetic complications [3]. Several studies have observed that patients with overweight and obesity have a significant positive correlation between BMI and oxidative stress biomarkers [4,5]. Oxidative stress in obesity can be caused by hyperglycaemia, chronic inflammation, endothelial dysfunction, vitamin and mineral deficiencies, impaired mitochondrial function, high tissue lipid levels, activity to carry excessive weight, and type of diet [6]. Oxidative stress is characterized by an excessive production of reactive oxygen species (ROS), which should be neutralized by antioxidant enzymes including catalase, superoxide dismutase (SOD), and glutathione peroxidase (GPx) and non-enzymatic molecules such as antioxidant vitamins, glutathione or polyphenols [7].

A possible cause of the obesity-induced pro-inflammatory state, based on the establishment of oxidative stress, is a high concentration of pro-inflammatory cytokines in serum in these patients [8]. TNF-α and IL-6 are cytokines secreted by adipocytes which are critical in the development of the inflammatory state associated with obesity [8]. Their concentration has been found to be related to the quantity and distribution of adipose tissue in the body [7]. Specifically, the elevated glucose and insulin concentration are parallel with the increase in IL-6, which suggests an involvement of the cytokine in glucose metabolism in adipocytes [8]. Moreover, it has been demonstrated that the increase in TNF-α expression in obese patients could be a key factor in limiting the extension of adipose tissue across the induction of insulin resistance in adipocytes [9].

Peripheral blood mononuclear cells (PBMCs), comprising lymphocytes and monocytes, are cells that play crucial roles in the immune system, and they also participate in pathological processes associated with obesity or metabolic syndrome (MetS) [10,11]. Accordingly, the expression of biomarkers with anti-inflammatory characteristics is reduced in PBMCs from obese people with respect to those from lean peers, suggesting that PBMCs may contribute to the inflammatory dysregulation in these patients [12]. Moreover, it has been evidenced that PBMCs from healthy elderly with an active lifestyle have better antioxidant defences and oxidative metabolism capabilities than sedentary peers [13]. Furthermore, it has been shown that patients with MetS have a systemic proinflammatory state which can result in the mobilization and activation of PBMC. This is supported by an increment of the antioxidant defences that may be derived from a pre-activation state of immune cells [11]. Altogether, the objective of the current study was to evaluate the oxidative stress and inflammatory biomarkers in plasma and PBMCS from adults with normal weight, overweight, and obesity. 

## 2. Materials and Methods 

### 2.1. Design and Participants

One hundred and fifty participants (55–80-years-old; 60% women) who were residents of the Balearic Islands were included in the study. This sex-age range was chosen because people are at high risk of overweight/obesity and related co-morbidities (type 2 diabetes mellitus, metabolic syndrome, and cardiovascular diseases) at this age.

The participants were distributed according to their body mass index (BMI): normal weight (18.5–24.9 kg/m^2^), overweight (25.0–29.9 kg/m^2^) and obese (more than 30.0 kg/m^2^) [14]. Patients were excluded when they were institutionalized, they demonstrated an inability to respond questionnaires due to physical or mental illness, alcoholism, drug addiction, or were taking clinical research drugs over past year. The Ethics Committee of Research of Balearic Islands (ref. CEIC-IB1295/09PI and CEIC-IB2251/14PI) approved the study protocols, following the Declaration of Helsinki. Participants were informed of the procedure and provided written informed consent.

### 2.2. Anthropometric and Physical Activity Characterization

To reduce the inter-observer coefficients of variation, the anthropometric variables were carried out by specialist observers. Height was determined with an anthropometer (Seca 214, SECA Deutschland, Hamburg, Germany) to the closest millimetre. A Segmental Body Composition Analyzer (Tanita BC-418, Tanita, Tokyo, Japan) was used to determine body weight and body fat, with subjects in light clothes and without shoes, and subtracting 0.6 kg for the clothes. BMI values were calculated as weight (kg)/height (m^2^). A validated semi-automatic oscillometer (Omron HEM, 705CP, Hoofddorp, The Netherlands) was used to measure blood pressure; measurements were made in the arm, registering the highest diastolic blood pressure, taking three measures after the participant was seated for 5 min, waiting for 1 min between each determination. An anthropometric tape was used to measure abdominal obesity, halfway between the last rib and the iliac crest. Waist-to-height ratio (WHtR) was calculated as waist (cm)/height (cm). The physical activities performed were evaluated as metabolic equivalents (METs) considering the rate of energy waste [15]. The participants reported the average weekly activities carried out in min/week.

### 2.3. Plasma and PBMCs Isolation 

Venous blood samples from all participants were obtained after a 12 h night-time fast in ethylene diamine tetraacetic acid (EDTA) sample tubes and were centrifuged at 1700× *g*, 15 min, 4°C. The protocol for the separation of white blood cells [16] was used to isolate PBMCs fraction from fresh whole blood using Ficoll-Paque PLUS (GE Healthcare Bio-Sciences AB, Uppsala, Sweden) and then centrifuged (900× *g*, 30 min, 4 °C). The final precipitate of PBMCs was washed with phosphate-buffered saline (PBS) at pH 7.4 and centrifuged at 900× *g*, for 10 min at 4 °C. 

### 2.4. Biochemical Parameters and Hemogram

Glucose, triglycerides, high-density lipoprotein (HDL) and total cholesterol were determined in plasma by standard procedures using standard enzymatic methods. Hemogram and haematological parameters were measured using an automatic flow cytometer analyser Technicon H2 (VCS system, Bayer, Leverkusen, Germany). 

### 2.5. Antioxidant Protein Levels

Western blot analysis was used to assess the levels of the PBMCs’ antioxidant enzyme proteins of all participants. In this process, 50 µg of protein from lysed cells was placed in an 15% SDS-polyacrylamide gel using Precision Plus Protein Kaleidoscope^TM^ (Bio-Rad) as a molecular weight marker. The electrophoresis was at 60 V for 15 min and then 150 V for 1 h. A Trans-Blot^®^ Turbo^TM^ Transfer System (Bio-Rad, Segrate, Milan, Italy) was used to electro-transfer the obtained bands onto a 0.2μm nitrocellulose membrane. After blocking the membranes with 5% non-fat powdered milk in TBS pH 7.5, 0.1% Tween 20, they were incubated overnight at 4 °C with the primary monoclonal antibodies. Antibodies anti-catalase (CAT) ref. 219010 (1:1000, rabbit polyclonal) and anti-Mn superoxide dismutase (MnSOD) ref. 574596 (1:1000, sheep polyclonal) (Merck KGaA, Darmstadt, Germany); anti-glutathione (GPx) ref. sc-22146 (1:200, goat polyclonal), anti-glutathione reductase (GRd) ref. sc-133136 (1:1000, mouse monoclonal) and actin ref. sc-8432, used as the loading control (1:200, mouse monoclonal) (Santa Cruz Biotechnology, CA, USA). A secondary peroxidase-conjugated antibody against specific primary antibody, for anti-CAT (ref. A0545) (1:5000), anti-MnSOD (ref. A3415) (1:5000) and anti-GRd (ref. A9044) (1:5000) and anti-GPx (ref. A5420) (1:10000) was used. Immunoblots were revealed by an enhanced chemiluminescence kit (Immun-Star^®^ Western C^®^ Kit reagent, Bio-Rad Laboratories, Hercules, CA, USA) to visualize the antibody-bound proteins. Signals were detected using a ChemiDoc XRS charge-coupled device imaging camera and quantified using the image analysis program Quantity One (Bio-Rad). Chemiluminescence exposure times to a film/CCD camera were varied over a period of 5–500 s to better resolve the different protein bands. Actin was used as a housekeeper protein to normalize Western blot bands for protein loading, since its expression was unaffected by the experimental treatments. It is considered a normal weight group with 100% of protein levels; therefore, the overweight and obese groups were recalculated with respect to it.

### 2.6. Enzymatic Determinations 

All the enzymatic determinations in PBMCs were determined using a Shimadzu UV-2100 spectrophotometer (Shimadzu Corporation, Kyoto, Japan) at 37 °C. Catalase (CAT) activity was analysed by the spectrophotometric method of Aebi based on the decomposition of H_2_O_2_ [17]. Superoxide dismutase (SOD) activity was measured using an adaptation of the method of McCord and Fridovich [18]. Glutathione peroxidase (GPx) activity was determined using a modification of the spectrophotometric method of Glohé and Gunzler [19]. Glutathione reductase (GRd) activity was measured by means of an adaptation of the Goldberg and Spooner spectrophotometric method [20]. The activity of CAT was expressed as k(s^−1^)/10^9^ cells and the other enzymatic activities as nkatal (nKat)/10^9^ cells. 

### 2.7. Malondialdehyde Assay 

A marker of lipid peroxidation, malondialdehyde (MDA) concentration (nM), was analysed in plasma and in PBMCs of all participants by a specific colorimetric assay kit (Sigma-Aldrich Merck^®^, St. Louis, MO, USA) following the manufacturer’s instructions [11]. 

### 2.8. PBMCs ROS Production

PBMCs ROS production was monitored after activation with lipopolysaccharide (LPS) from *Escherichia coli* (Sigma-Aldrich, St. Louis, MO, USA). Cell suspensions (6 × 10^5^ cells) were introduced in a 96-well microplate and LPS prepared in 2 mM in phosphate buffer saline, pH 7.4 was also added to the wells. Then, the cell-permeant probe 2,7-dichlorofluorescein-diacetate (DCFH-DA, 61.6 µM in Hanks’ Balanced Salts Medium) was added to all wells. The fluorescence (Ex, 480 nm; Em, 530 nm) was recorded at 37 °C for 60 min in FLx800 Microplate Fluorescence Reader (Bio-tek Instruments, Inc., Winuschi, VT, USA).

### 2.9. Cytokine Assays

Cytokine (TNFα and IL-6) levels were determined in plasma of all participants using individual ELISA kits (Diaclone, Besancon Cedex, France) following the manufacturer’s instructions for use. The intra- and inter-assay coefficients of variation were 3.2% and 10.9% for TNFα and 4.4% and 9.1% for IL-6, respectively. 

### 2.10. Statistics

Statistical analysis was carried out with the Statistical Package for Social Sciences (SPSS v.25, IBM Software Group, Chicago, IL, USA). Continuous variables were presented as means ± standard error of the mean (SEM), and considering *p* < 0.05 as statistically significant. Kolmogorov–Smirnov test was used to assess normality of data. One-way analysis of variance (ANOVA) after adjustment for BMI was used to check significance of data. Bonferroni post hoc test was carried out when significant differences were found between groups. A receiver operating characteristic (ROC) curve and area under the curve (AUC) was used to analyse the biomarker results.

## 3. Results

### 3.1. Anthropometric and Haematological Parameters

Table 1 shows the anthropometric characteristics of the participants stratified by BMI. Glucose levels, triglycerides levels, abdominal obesity, and WHtR were significantly higher, whereas HDL-cholesterol was significantly lower in the obese group. The differences between HDL-cholesterol, abdominal obesity, and WHtR were also significant in the overweight group in comparison to the normal-weight group. No significant differences between groups were observed in systolic and diastolic blood pressure probably due to taking antihypertensive drugs: 77% of the obese group, 40% of the overweight group, and 13% of the normal-weight group. In addition, 36% of obese participants and 18% of overweight patients were taking hyperglycaemia medication. Total physical activity (MET·hour/week) was reduced significantly in the obese group compared to the normal-weight group. 

Haematological parameters of participants are presented in Table 2. No significant differences were evidenced in haematocrit, erythrocyte, lymphocytes, and eosinophils counts between the three groups. The number of leukocytes and monocytes were significantly higher in the obese group in contrast to the other groups. Neutrophils and basophils were significantly increased in the obese group compared to the normal-weight group.

### 3.2. Biomarkers of Oxidative Stress 

**Figure 1:**
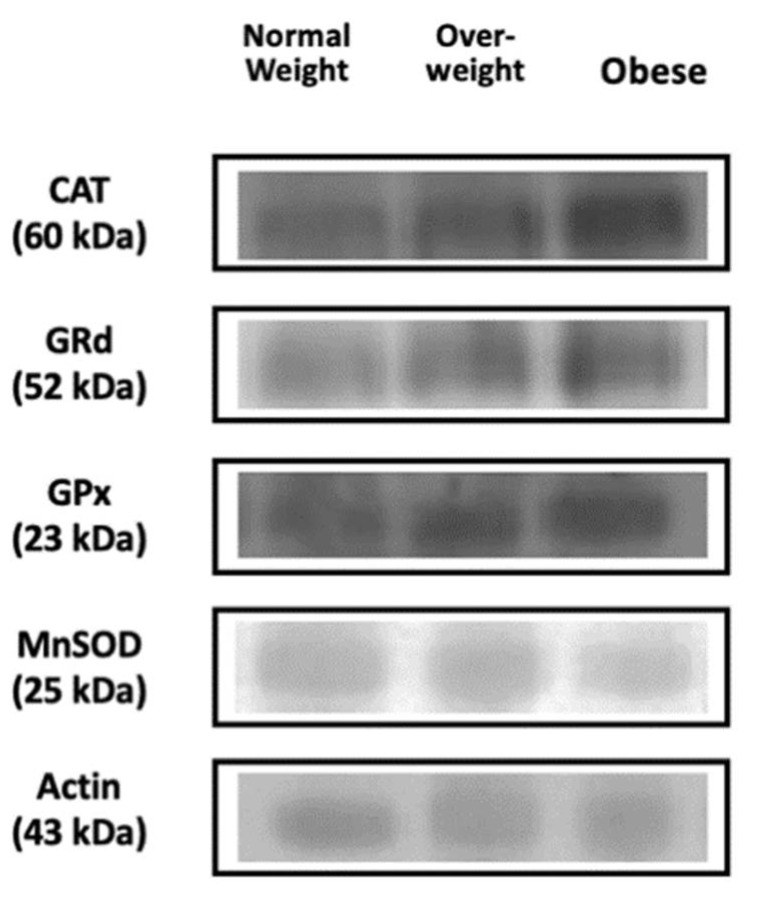
Representative bands of Western blots of CAT, GRd, GPx, MnSOD and actin in PBMCs (*n* = 50 each group).

The activities of the antioxidant enzymes in PBMCs are shown in Table 3. Catalase, GRd and SOD activities were significantly higher in the obese and overweight groups compared to the normal-weight group. GPx activity was significantly higher in the obese group. The results of protein levels in PBMCs of CAT, GPX, GRd and mnSOD are shown in Table 3 and Figure 1. No significant differences were evidenced in GPx and MnSOD protein levels.

**Figure 2:**
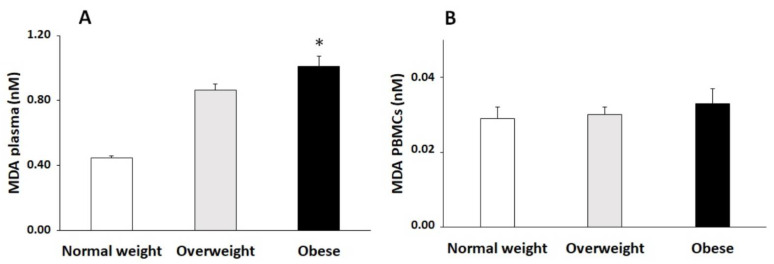
MDA levels (mean ± SEM) in plasma (**A**) and in PBMCs (**B**) classified according to BMI (*n* = 50 each group). Statistical analysis by one-way ANOVA. * Differences regarding normal-weight participants.

Plasma and PBMCs MDA levels, a marker of lipid peroxidation, were shown in Figure 2a,b, respectively. The obtained data reported significantly higher levels in plasma MDA from the obese group with respect to the normal-weight group, whereas no significant differences were observed in PBMCs.

**Figure 3:**
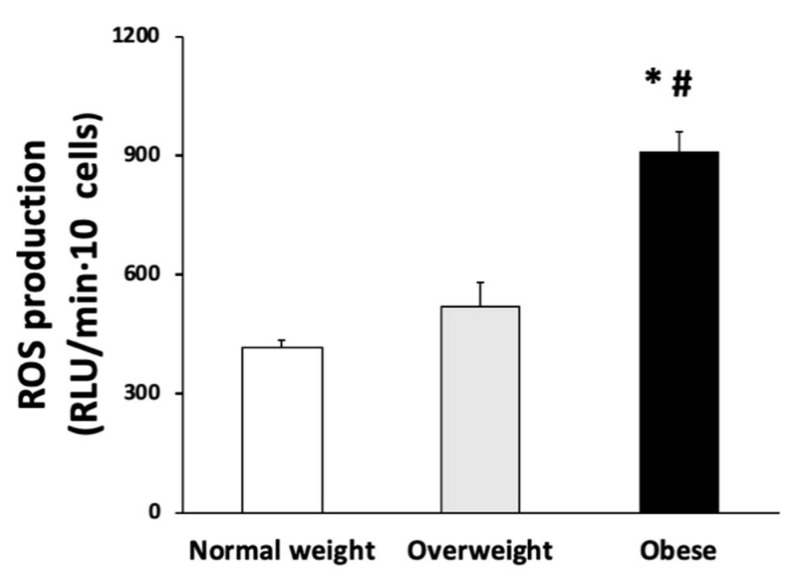
ROS production in PBMCs stimulated with LPS were classified according to BMI (*n* = 50 each group). Statistical analysis by one-way ANOVA. * Difference in means between participants with normal weight. # Differences regarding overweight participants.

The ROS production by PBMCs stimulated with LPS is presented in Figure 3. The production of ROS was progressively increasing with overweight and obesity, with significant differences in the normal-weight and overweight groups when compared with the obese group.

### 3.3. Cytokine Levels

**Figure 4:**
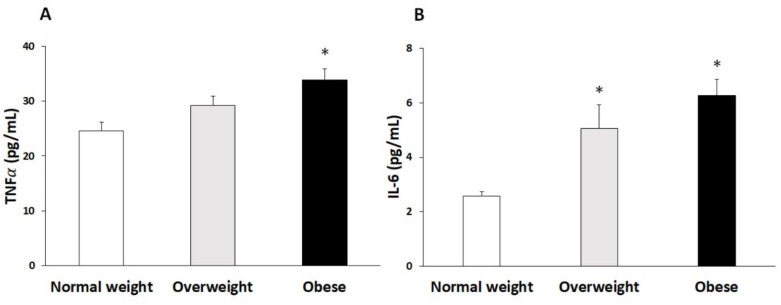
TNFα levels (**A**) and IL-6 levels (**B**) in plasma (mean ± SEM) classified according to BMI (*n* = 50 each group). Statistical analysis by one-way ANOVA. * Differences regarding normal-weight participants.

The plasma proinflammatory cytokine concentration is presented in Figure 4a,b, respectively. Levels of TNFα were significantly higher in the obese group compared to the normal-weight group, while the levels of IL-6 were significantly higher in the obese and overweight group with respect to the normal-weight group.

### 3.4. ROC Curve of Biomarkers According to BMI

**Figure 5:**
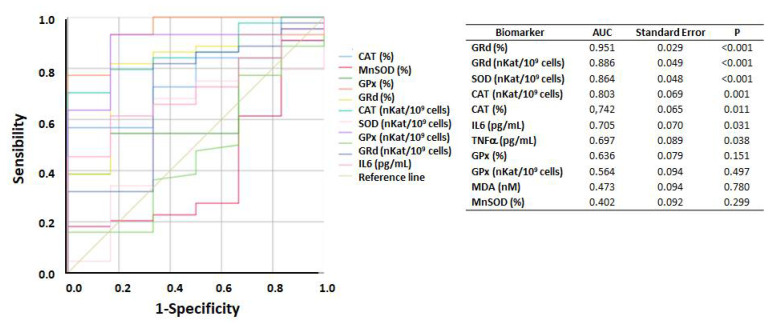
ROC curve of the accuracy of markers in assessing BMI. AUC, area under the curve; CAT, catalase; GPx, glutathione peroxidase; GRd, Glutathione reductase; IL-6, Interleukin 6; MDA, malondialdehyde; MnSOD, manganese superoxide dismutase; SOD, superoxide dismutase; TNFα: tumour necrosis factor alpha. (%): protein levels; (nKat/109 cells): activity. *p* values by: asymptotic significance.

Figure 5 illustrates the accuracy of PBMCs markers in assessing BMI using a ROC curve (BMI > 25 vs. BMI ≤ 25). The best and significative area under the curve (AUC) was found for GRd (protein level and activity), SOD (protein level), CAT (protein level and activity), IL6 and TNFα, representing 96%, 89%, 86%, 80%, 74%, 70%, and 70% of the AUC over the reference line, respectively, showing that they are good high BMI predictors.

## 4. Discussion

The main findings of the current study are the progressive increase in the number of circulating leukocytes, and the degree of oxidative stress and pro-inflammatory biomarkers in PBMCs and plasma, which occurs with overweight and obesity. The higher number of leukocytes, neutrophils, basophils, and monocytes observed in obese participants when compared with normal-weight peers is probably related to the proinflammatory state associated with this disorder. Previous studies suggested that the mobilization of immune cells and the release of proinflammatory mediators can be caused by the inflammation of adipose tissue due to overweight and obesity [21,22]. Specifically, it has been evidenced that BMI, body fat, and insulin resistance are positively correlated with the number of circulating neutrophils, monocytes, and lymphocytes [23]. In this sense, previous evidence reported that macrophages are not the only immune cell type that can be accumulated into the obese adipose tissue [24]. Different subsets of lymphocytes and neutrophils have also been observed within adipose tissue, which can modify macrophage behaviour, favour inflammation and promote insulin resistance [25,26]. 

Obese subjects from the present study also showed higher plasma levels of the pro-inflammatory TNFα and IL-6 with respect to normal-weight peers. Obese subjects have a persistent low-grade inflammatory state which alters adipocyte metabolism and gene expression, increasing lipolysis and the release of mediators that recruit and activate macrophages such as TNFα, IL-6, IL-1β and monocyte chemotactic protein-1 (MCP-1) [8,24]. These cytokines are mainly synthesized by adipocytes, but they can also be secreted by endothelial cells, fibroblasts, immune cells and even skeletal muscle [27]. Thus, the concentration of TNFα and IL-6 is related to the distribution and percentage of adipose tissue, with higher production from visceral adipose tissue than from subcutaneous adipose tissue [8]. 

Moreover, current participants also showed a progressive increase in abdominal obesity and WHtR that could be responsible for the increase in circulating levels of both pro-inflammatory cytokines. The present results confirm that obese participants had chronic inflammation due to an increase in circulating inflammatory cytokines that can contribute to mobilize and pre-activate the immune cells, mainly neutrophils and monocytes. In addition, higher plasma levels of MDA were also found in obese subjects than in normal-weight and overweight participants. MDA is a widely used marker of oxidative damage as well as the main end-product of lipid peroxidation [28]. The obtained results are in accordance with previous studies which described that plasma MDA values were elevated in the obese subjects and can be reduced with weight loss [5]. It has been previously reported that obese people showed low intake of antioxidant-rich foods compared to non-obese people, which leads to lower levels of antioxidants in the blood [29,30]. Additionally, the protective antioxidant enzymes including SOD and GPx are depleted in chronic obese subjects [31,32]. MDA in conjunction with transition metals can generate more ROS, resulting in a cycle of ROS formation and oxidative stress that contributes to impaired glucose metabolism [33]. 

The increased activity and protein levels of antioxidant enzymes in PBMCs observed in overweight and obese subjects from the present study may be a consequence of a pre-activation state of these cells, which is a derivative of chronic inflammation and oxidative stress-related to excessive fat accumulation [11,34]. In this sense, the activation of PBMCs with LPS as an immunomodulator follows the same response pattern, increasing in parallel with the BMI and suggesting the existence of a pre-activation state of these cells. Although PBMCs are not the main source of pro-inflammatory mediators in obesity, it has been suggested that the gene expression profile of these cells may reflect the amount of visceral fat and, consequently, be indicative of the inflammatory state in obesity [35,36]. It has been reported that PBMCs from obese participants exhibit a higher proinflammatory secretory profile than normal-weight subjects with increased production of TNFα and IL-2, and lower production of the anti-inflammatory cytokine IL-10 [35,37,38]. In fact, the exposure of PBMCs to H_2_O_2_ induces the expression of Toll-like receptors (TLRs) 2 and 4 that activate pro-inflammatory signalling pathways such as the nuclear factor κB pathway (NFκB) pathway [39]. In addition to oxidative stress, the activation of these pro-inflammatory pathways is increased by the increased presence of fatty acids in obese subjects and by the action of LPS [39,40]. Thus, the pro-inflammatory and pro-oxidant situation of PBMCs could induce the observed increase in antioxidant enzymes to adapt to and compensate for the new demanding situation. Furthermore, the fact that obese subjects showed higher levels of oxidative stress markers in plasma, such as MDA, 3-nitrotyrosine, or advanced oxidation protein products and pro-oxidant enzymes such as myeloperoxidase (MPO) or xanthine oxidase (XO) may also contribute to the induction of antioxidant defence mechanisms in PBMCs [41,42]. Increased plasma protein levels of MPO and XO were observed in obese patients according to the increase in the degree of intrahepatic fat accumulation [43]. Accordingly, we previously showed that ex vivo treatment for 2 h with H_2_O_2_ (produced with glucose oxidase) in PBMCs from patients with metabolic syndrome to mimic chronic stress caused a transient inflammatory response with an increase in the production of IL6, IL8, and IL1β [44]. The observed increase in the activities of antioxidant enzymes in PBMCs associated with overweight and obesity is not related to an increase in MDA levels, showing that PBMCs can maintain their oxidative status, even in obese patients. However, it cannot be ruled out that in a situation where the degree of stress increases, the PBMCs of obese subjects are more susceptible to oxidative damage. In this sense, it has been observed that PBMCs from obese and diabetic subjects exposed in vitro to adrenaline show greater DNA and cell membrane damage [45]. On the other hand, weight reduction in obese patients after an intervention with a hypocaloric diet for 8 weeks decreased the expression of genes in PBMCs related to oxidative stress, such as NADH-coenzyme Q reductase (NDUFS2), and inflammation, such as IL-8, receptor-interacting serine-threonine kinase (RIPK3) and TNF-α induced protein 3 interacting protein 1 (TNIP1) [46]. Moreover, significant differences in total comet score (TCS) between PBMCs of normal weight and obese participants have been observed when a higher concentration of adrenaline induced DNA damage in the obese, prediabetic and diabetic patients [45]. 

As was expected, current obese patients also showed a higher body weight, BMI, abdominal obesity, WHtR, glucose and triglyceride levels, and lesser HDL-cholesterol than normal-weight patients, in accordance with previous studies reporting that an increase in visceral abdominal fat volume is proportionally correlated with an increase in WHtR and BMI in overweight and obesity, and these patients have a major incremental on the cardio-metabolic risk factors [2,7].

The present results from the ROC curve and area under the curve show that the PBMCs’ levels of SOD, GRd, CAT, IL6, and TNFα may be good BMI markers with potential utility in clinical practice.

## 5. Strengths and Limitations

The main strength of this study is that an association has been found between plasma and PBMC oxidative stress and proinflammatory biomarker levels and normal weight, overweight, and obese stages. The main limitation of this study is that sample size was relatively reduced; nevertheless, the sample size was enough to evidence the existence of differences in the biomarker levels between normal-weight, overweight, and obese participants in the study.

## 6. Conclusions

The current study has demonstrated a progressive increase in oxidative stress and inflammation among overweight and obese individuals when compared with normal-weight subjects. Plasma oxidative stress and inflammation biomarkers were mainly increased in obese subjects. Nevertheless, in PBMCs, the antioxidant defences were seen to increase as a compensatory mechanism in the face of a chronic pro-oxidative and pro-inflammatory stimulus situation. The relationship between the biomarkers studied and overweight/obesity suggests that they may be useful for monitoring the progression/reversal of obesity.

## Figures and Tables

**Table 1:** Characteristics of participants stratified by BMI.

	Normal Weight (*n* = 50)	Overweight (*n* = 50)	Obese (*n* = 50)	*p*-Value
Age (years)	65.6 ± 0.7	64.9 ± 0.7	64.0 ± 0.6	0.246
Weight (kg)	61.3 ± 1.1	74.5 ± 1.4 *	92.9 ± 1.8 *#	<0.001
Height (cm)	161.9 ± 1.1	163.7 ± 1.4	162.3 ± 1.3	0.586
BMI (kg/m^2^)	23.0 ± 0.2	27.6 ± 0.2 *	35.2 ± 0.4 *#	<0.001
Total physical activity (MET·hour/week)	66.6 ± 3.2	51.9 ± 4.0	46.9 ± 5.4 *	0.007
Systolic blood pressure (mmHg)	136.8 ± 3.5	135.7 ± 2.3	135.2 ± 3.7	0.941
Diastolic blood pressure (mmHg)	78.3 ± 1.5	77.5 ± 1.3	76.9 ± 1.9	0.838
Glucose (mg/dL)	91.3 ± 1.0	101.2 ± 2.3	118.7 ± 4.8 *#	<0.001
Triglycerides (mg/dL)	91.6 ± 4.2	102.0 ± 5.1	154.5 ± 8.9 *#	<0.001
HDL-cholesterol (mg/dL)	61.5 ± 1.7	51.6 ± 1.4 *	44.4 ± 1.3 *#	<0.001
Abdominal obesity (cm)	82.1 ± 1.6	93.7 ± 1.2 *	115.3 ± 1.3 *#	<0.001
WHtR	0.51 ± 0.01	0.57 ± 0.01 *	0.71 ± 0.01 *#	<0.001

Results are expressed as mean ± SEM. Abbreviations: BMI, body mass index; MET, metabolic equivalent of task; SEM, standard error of media; WHtR, waist to height ratio. Statistical analysis by one-way ANOVA. * Differences regarding normal-weight participants. # Differences regarding overweight participants.

**Table 2:** Hematological parameters of participants stratified by BMI.

	Normal Weight (*n* = 50)	Overweight (*n* = 50)	Obese (*n* = 50)	ANOVA
Hematocrit (%)	43.0 ± 0.5	43.7 ± 0.5	42.6 ± 0.59	0.367
Erythrocytes (10^6^/mm^3^)	4.74 ± 0.06	4.79 ± 0.06	4.71 ± 0.07	0.715
Leukocytes (10^3^/mm^3^)	6.39 ± 0.21	6.57 ± 0.25	7.45 ± 0.25 *#	0.004
Neutrophils (10^3^/mm^3^)	3.27 ± 0.16	3.57 ± 0.19	4.09 ± 0.16 *	0.004
Lymphocytes (10^3^/mm^3^)	2.36 ± 0.10	2.23 ± 0.09	2.45 ± 0.12	0.342
Basophils (10^3^/mm^3^)	0.04 ± 0.00	0.048 ± 0.00	0.050 ± 0.00 *	0.026
Monocytes (10^3^/mm^3^)	0.48 ± 0.02	0.545 ± 0.03	0.633 ± 0.03 *#	<0.001
Eosinophils (10^3^/mm^3^)	0.24 ± 0.03	0.18 ± 0.02	0.23 ± 0.02	0.150

Results are expressed as mean ± SEM. Abbreviations: BMI, body mass index; SEM, standard error of media. Statistical analysis by one-way ANOVA. * Differences regarding normal-weight participants. # Differences regarding overweight participants.

**Table 3:** PBMCs enzymatic activities and protein levels of participants stratified by BMI.

	Normal Weight (*n* = 50)	Overweight (*n* = 50)	Obese (*n* = 50)	ANOVA
**PBMCs activity**				
CAT (K/10^9^ cells)	10.8 ± 2.2	25.4 ± 3.1 *	34.9 ± 3.8 *	<0.001
GRd (nkat/10^9^ cells)	221 ± 46	305 ± 37 *	448 ± 34 *	<0.001
GPx (nkat/10^9^ cells)	53.4 ± 6.1	57.1 ± 5.4	73.7 ± 4.7 *#	0.017
SOD (nkat/10^9^ cells)	39.3 ± 4.8	59.3 ± 5.5 *	74.7 ± 4.4 *	<0.001
**PBMCs protein levels**				
CAT (%)	100 ± 12	127 ± 8	140 ± 8 *	0.015
GRd (%)	100 ± 16	157 ± 14 *	169 ± 13 *	0.009
GPx (%)	100 ± 8	116 ± 6	118 ± 8	0.307
MnSOD (%)	100 ± 11	84.3 ± 9.8	77.7 ± 11.4	0.396

Results (*n* = 50 each group) are expressed as mean ± SEM. Abbreviations: BMI, body mass index; CAT, catalase; SOD, superoxide dismutase; MPO, myeloperoxidase; GRd, glutathione reductase.; GPx, glutathione peroxidase. mnSOD, mitocondrial antioxidant manganese superoxide dismutase. Statistical analysis performed using one-way ANOVA. * Differences regarding normal-weight participants. # Differences regarding overweight participants.

## Data Availability

There are restrictions on the availability of data for this trial, due to the signed consent agreements around data sharing, which only allow access to external researchers for studies following the project purposes. Requestors wishing to access the trial data used in this study can make a request to pep.tur@uib.es.

## References

[1] Biswas T., Garnett S.P., Pervin S., Rawal L.B. (2017). The prevalence of underweight, overweight and obesity in Bangladeshi adults: Data from a national survey. PLoS ONE.

[2] Pahwa R., Adams-Huet B., Jialal I. (2017). The effect of increasing body mass index on cardio-metabolic risk and biomarkers of oxidative stress and inflammation in nascent metabolic syndrome. J. Diabetes Complicat..

[3] Skalicky J., Muzakova V., Kandar R., Meloun M., Rousar T., Palicka V. (2008). Evaluation of oxidative stress and inflammation in obese adults with metabolic syndrome. Clin. Chem. Lab. Med..

[4] Ataey A., Jafarvand E., Adham D., Moradi-Asl E. (2020). The Relationship Between Obesity, Overweight, and the Human Development Index in World Health Organization Eastern Mediterranean Region Countries. J. Prev. Med. Public Health.

[5] Vincent H.K., Taylor A.G. (2006). Biomarkers and potential mechanisms of obesity-induced oxidant stress in humans. Int. J. Obes..

[6] Manna P., Jain S.K. (2015). Obesity, Oxidative Stress, Adipose Tissue Dysfunction, and the Associated Health Risks: Causes and Therapeutic Strategies. Metab. Syndr. Relat. Disord..

[7] García-Sánchez A., Gámez-Nava J.I., Díaz-de la Cruz E.N., Cardona-Muñoz E.G., Becerra-Alvarado I.N., Aceves-Aceves J.A., Sánchez-Rodríguez E.N., Miranda-Díaz A.G. (2020). The Effect of Visceral Abdominal Fat Volume on Oxidative Stress and Proinflammatory Cytokines in Subjects with Normal Weight, Overweight and Obesity. Diabetes Metab. Syndr. Obes. Targets Ther..

[8] Popko K., Gorska E., Stelmaszczyk-Emmel A., Plywaczewski R., Stoklosa A., Gorecka D., Pyrzak B., Demkow U. (2010). Proinflammatory cytokines IL-6 and TNF-α and the development of inflammation in obese subjects. Eur. J. Med. Res..

[9] Cawthorn W.P., Sethi J.K. (2008). TNF-α and adipocyte biology. FEBS Lett..

[10] Jung U.J., Seo Y.R., Ryu R., Choi M.S. (2016). Differences in metabolic biomarkers in the blood and gene expression profiles of peripheral blood mononuclear cells among normal weight, mildly obese and moderately obese subjects. Br. J. Nutr..

[11] Monserrat-Mesquida M., Quetglas-Llabrés M., Capó X., Bouzas C., Mateos D., Pons A., Tur J.A., Sureda A. (2020). Metabolic Syndrome is Associated with Oxidative Stress and Proinflammatory State. Antioxidants.

[12] Bories G., Caiazzo R., Derudas B., Copin C., Raverdy V., Pigeyre M., Pattou F., Staels B., Chinetti-Gbaguidi G. (2012). Impaired alternative macrophage differentiation of peripheral blood mononuclear cells from obese subjects. Diabetes Vasc. Dis. Res..

[13] Busquets-Cortés C., Capó X., del Mar Bibiloni M., Martorell M., Ferrer M.D., Argelich E., Bouzas C., Carreres S., Tur J.A., Pons A. (2018). Peripheral blood mononuclear cells antioxidant adaptations to regular physical activity in elderly people. Nutrients.

[14] Report of a who Expert Committee (1995). Physical status: The use and interpretation of anthropometry. World Health Organ. Tech. Rep. Ser..

[15] Ainsworth B.E., Haskell W.L., Leon A.S., Jacobs D.R., Montoye H.J., Sallis J.F., Paffenbarger R.S. (1993). Compendium of Physical Activities: Classification of energy costs of human physical activities. Med. Sci. Sport. Exerc..

[16] Boyum A. (1964). Separation of White Blood Cells. Nature.

[17] Aebi H. (1984). Catalase in vitro. Methods Enzymol..

[18] McCord J.M., Fridovich I. (1969). Superoxide dismutase. An enzymic function for erythrocuprein (hemocuprein). J. Biol. Chem..

[19] Glohé L., Gunzler W. (1984). Assay of glutathione peroxidase. Methods Enzymol..

[20] Bergmayer H.U. (1963). Glutathione reductase. Methods of Enzymatic Analysis. Starch Stärke.

[21] Nihi M.M., Manfro R.C., Martins C., Suliman M., Murayama Y., Riella M.C., Lindholm B., Nascimento M.M. (2010). do [Association between body fat, inflammation and oxidative stress in hemodialysis]. J. Bras. Nefrol..

[22] Sureda A., Bibiloni M., Julibert A., Bouzas C., Argelich E., Llompart I., Pons A., Tur J. (2018). Adherence to the Mediterranean Diet and Inflammatory Markers. Nutrients.

[23] Ryder E., Diez-Ewald M., Mosquera J., Fernández E., Pedreañez A., Vargas R., Peña C., Fernández N. (2014). Association of obesity with leukocyte count in obese individuals without metabolic syndrome. Diabetes Metab. Syndr. Clin. Res. Rev..

[24] Olefsky J.M., Glass C.K. (2009). Macrophages, inflammation, and insulin resistance. Annu. Rev. Physiol..

[25] Rocha V.Z., Folco E.J., Sukhova G., Shimizu K., Gotsman I., Vernon A.H., Libby P. (2008). Interferon-γ, a Th1 cytokine, regulates fat inflammation: A role for adaptive immunity in obesity. Circ. Res..

[26] Elgazar-Carmon V., Rudich A., Hadad N., Levy R. (2008). Neutrophils transiently infiltrate intra-abdominal fat early in the course of high-fat feeding. J. Lipid Res..

[27] Gutiérrez-Ruiz J., Velázquez-Paniagua M., Prieto-Gómez B. (2011). El tejido adiposo como órgano maestro en el metabolismo. Rev. Endocrinol. Nutr..

[28] Adnan M.T., Amin M.N., Uddin M.G., Hussain M.S., Sarwar M.S., Hossain M.K., Uddin S.M.N., Islam M.S. (2019). Increased concentration of serum MDA, decreased antioxidants and altered trace elements and macro-minerals are linked to obesity among Bangladeshi population. Diabetes Metab. Syndr. Clin. Res. Rev..

[29] Vincent H.K., Innes K.E., Vincent K.R. (2007). Oxidative stress and potential interventions to reduce oxidative stress in overweight and obesity. Diabetes Obes. Metab..

[30] Galan P., Viteri F.E., Bertrais S., Czernichow S., Faure H., Arnaud J., Ruffieux D., Chenal S., Arnault N., Favier A. (2005). Serum concentrations of β-carotene, vitamins C and E, zinc and selenium are influenced by sex, age, diet, smoking status, alcohol consumption and corpulence in a general French adult population. Eur. J. Clin. Nutr..

[31] Ozata M., Mergen M., Oktenli C., Aydin A., Sanisoglu S.Y., Bolu E., Yilmaz M.I., Sayal A., Isimer A., Ozdemir I.C. (2002). Increased oxidative stress and hypozincemia in male obesity. Clin. Biochem..

[32] Olusi S.O. (2002). Obesity is an independent risk factor for plasma lipid peroxidation and depletion of erythrocyte cytoprotectic enzymes in humans. Int. J. Obes..

[33] Yue K.K.M., Chung W.S., Leung A.W.N., Cheng C.H.K. (2003). Redox changes precede the occurrence of oxidative stress in eyes and aorta, but not in kidneys of diabetic rats. Life Sci..

[34] Ladeiras-Lopes R., Teixeira P., Azevedo A., Leite-Moreira A., Bettencourt N., Fontes-Carvalho R. (2020). Metabolic syndrome severity score is associated with diastolic dysfunction and low-grade inflammation in a community-based cohort. Eur. J. Prev. Cardiol..

[35] O’Rourke R.W., Kay T., Lyle E.A., Traxler S.A., Deveney C.W., Jobe B.A., Roberts C.T., Marks D., Rosenbaum J.T. (2006). Alterations in peripheral blood lymphocyte cytokine expression in obesity. Clin. Exp. Immunol..

[36] Ghanim H., Aljada A., Hofmeyer D., Syed T., Mohanty P., Dandona P. (2004). Circulating mononuclear cells in the obese are in a proinflammatory state. Circulation.

[37] Catalán V., Gómez-Ambrosi J., Rodríguez A., Ramírez B., Valentí V., Moncada R., Silva C., Salvador J., Frühbeck G. (2015). Peripheral mononuclear blood cells contribute to the obesity-associated inflammatory state independently of glycemic status: Involvement of the novel proinflammatory adipokines chemerin, chitinase-3-like protein 1, lipocalin-2 and osteopontin. Genes Nutr..

[38] Dicker D., Salook M.A., Marcoviciu D., Djaldetti M., Bessler H. (2013). Role of peripheral blood mononuclear cells in the predisposition of obese individuals to inflammation and infection. Obes. Facts.

[39] Akhter N., Madhoun A., Arefanian H., Wilson A., Kochumon S., Thomas R., Shenouda S., Al-Mulla F., Ahmad R., Sindhu S. (2019). Oxidative Stress Induces Expression of the Toll-Like Receptors (TLRs) 2 and 4 in the Human Peripheral Blood Mononuclear Cells: Implications for Metabolic Inflammation. Cell. Physiol. Biochem..

[40] Bondia-Pons I., Ryan L., Martinez J.A. (2012). Oxidative stress and inflammation interactions in human obesity. J. Physiol. Biochem..

[41] Bankoglu E.E., Gerber J., Kodandaraman G., Seyfried F., Stopper H. (2020). Influence of bariatric surgery induced weight loss on oxidative DNA damage. Mutat. Res. Genet. Toxicol. Environ. Mutagen..

[42] Qaddoumi M.G., Alanbaei M., Hammad M.M., Al Khairi I., Cherian P., Channanath A., Thanaraj T.A., Al-Mulla F., Abu-Farha M., Abubaker J. (2020). Investigating the Role of Myeloperoxidase and Angiopoietin-like Protein 6 in Obesity and Diabetes. Sci. Rep..

[43] Monserrat-Mesquida M., Quetglas-Llabrés M., Abbate M., Montemayor S., Mascaró C.M., Casares M., Tejada S., Abete I., Zulet M.A., Tur J.A. (2020). Oxidative stress and pro-inflammatory status in patients with non-alcoholic fatty liver disease. Antioxidants.

[44] Busquets-Cortés C., Capó X., Argelich E., Ferrer M.D., Mateos D., Bouzas C., Abbate M., Tur J.A., Sureda A., Pons A. (2018). Effects of micromolar steady-state hydrogen peroxide exposure on inflammatory and redox gene expression in immune cells from humans with metabolic syndrome. Nutrients.

[45] Djelić N., Radaković M., Borozan S., Dimirijević-Srećković V., Pajović N., Vejnović B., Borozan N., Bankoglu E.E., Stopper H., Stanimirović Z. (2019). Oxidative stress and DNA damage in peripheral blood mononuclear cells from normal, obese, prediabetic and diabetic persons exposed to adrenaline in vitro. Mutat. Res. Genet. Toxicol. Environ. Mutagen..

[46] Crujeiras A.B., Parra D., Milagro F.I., Goyenechea E., Larrarte E., Margareto J., Martínez J.A. (2008). Differential expression of oxidative stress and inflammation related genes in peripheral blood mononuclear cells in response to a low-calorie diet: A nutrigenomics study. OMICS J. Integr. Biol..

